# Association between adverse pregnancy outcomes and preceding risk factors: a cross-sectional study from Nashik District, India

**DOI:** 10.1186/s12884-021-04174-w

**Published:** 2021-10-18

**Authors:** Prakash Prabhakarrao Doke, Sonali Hemant Palkar, Jayashree Sachin Gothankar, Archana Vasantrao Patil, Amruta Paresh Chutke, Prasad Dnyandeo Pore, Aniruddha Vinayakrao Deshpande, Khanindra Kumar Bhuyan, Madhusudan Vamanrao Karnataki, Aparna Nishikant Shrotri

**Affiliations:** 1 Department of Community Medicine, Bharati Vidyapeeth (DTU) Medical College, Pune-Satara Road, Pune, Maharashtra 411043 India; 2 State Family Welfare Bureau, Department of Public Health, Government of Maharashtra, Pune, India; 3 UNICEF Maharashtra, Mumbai, India

**Keywords:** Stillbirth, Abortion, Low birth weight, Congenital physical defect, Adverse pregnancy outcomes, Risk factors

## Abstract

**Background:**

The preconception phase of women’s life cycle is critical but comparatively ignored. The presence of health risks is judged as hazardous to the wellbeing of women and their offspring. This study aimed to estimate the prevalence of various pregnancy outcomes and assess the association between certain risk factors and adverse outcomes.

**Methods:**

As a part of a preconception care intervention project, a baseline survey was conducted in four blocks of Nashik District, India. In this population-based cross-sectional analytical study, we compared cases in the study group (randomly selected one tribal and one non-tribal block) with those of the control group (one tribal and one non-tribal block). A comparison was also made between the tribal and non-tribal blocks in each group. All women who had a pregnancy outcome in the preceding 12 months (01 April 2017 to 31 March 2018) were interviewed. Trained Accredited Social Health Activists conducted the survey under the direct supervision of Auxiliary Nurse Midwives and Medical Officers. Multivariate analysis was carried out to find the adjusted prevalence ratio of having a particular adverse outcome because of the prespecified potential risk factors.

**Results:**

A total of 9307 women participated in the study. The prevalence of adverse pregnancy outcomes was as follows: abortion in 4.1%, stillbirth in 1.7%, preterm birth in 4.1%, low birth weight in 13.2%, and congenital physical defect in 2.8%. Prevalence of parental consanguinity, pre-existing maternal illness at conception, heavy work during the last six months of pregnancy, tobacco consumption, alcohol consumption, direct exposure to pesticides and domestic violence during pregnancy was 18.5, 2.2, 18.7, 5.6, 0.5, 2.3, and 0.8% respectively. Risk factors associated with abortion included pre-existing illness and heavy work in the last six months of the pregnancy. Consanguinity, tobacco consumption during pregnancy and pre-existing illness were identified as risk factors for stillbirth. Significant risk factors of low birth weight were heavy work in the last six months of pregnancy, pre-existing illness and residence in a tribal area.

**Conclusion:**

There is a need to emphasize on maternal behaviour, including tobacco consumption, and heavy work during pregnancy, as well as on parental consanguinity and pre-existing maternal illnesses, in order to achieve the best possible pregnancy outcomes.

## Background

India is the second-largest country globally, and almost 70% of its population resides in the rural area. The urban-rural gap is evident through higher child mortality indicators for the rural area, which may result from the lack of health care facilities, apart from the socio-cultural environment [1, 2]. The challenges increase further for the tribal people, who constitute 8.6% of India’s total population [3]. This socio-culturally different, underprivileged community is dissociated from the health care system. The compromised availability and access to health care leads to poor utilisation of Maternal and Child Health services [4]. Due to many adversities, the health indicators of the tribal population are lower compared to non-tribal [5]. Preconception care (PCC) prevents mortality and morbidity among mothers and children [6]. Although the WHO [7] and the Government of India through the India Newborn Action Plan (INAP) [8], as well as the Federation of Obstetric and Gynaecological Associations of India, recommended the roll-out of PCC in India [9], it has not yet been rolled out systematically in many countries, including India.

PCC interventions were planned in the tribal and non-tribal rural blocks of Nashik district of Maharashtra, India. A baseline survey was conducted before the interventions with the following objectives:

1. To estimate rates of adverse pregnancy outcomes (abortion, stillbirth, preterm, low birth weight, and congenital physical defect) in Nashik district, India.
2. To compare the estimates between the study and control blocks, as well as between tribal and non-tribal blocks.
3. To assess the association between risk factors (parental consanguinity, heavy work in the last six months of pregnancy, tobacco consumption, alcohol consumption, exposure to the pesticide, domestic violence, pre-existing maternal illness at conception and residence in the tribal area) and adverse pregnancy outcomes.

## Methods

### Study design

This was a population-based cross-sectional analytical study conducted before the initiation of the PCC interventions.

### Study setting

With the support of UNICEF, the government of Maharashtra conducted a study in the rural and tribal areas of Nashik district to assess the effect of an intervention in the form of PCC on pregnancy outcomes. The study area included four blocks of Nashik district which were divided into a study group where the intervention was implemented and a control group where no such activity was conducted.

**Fig. 1:**
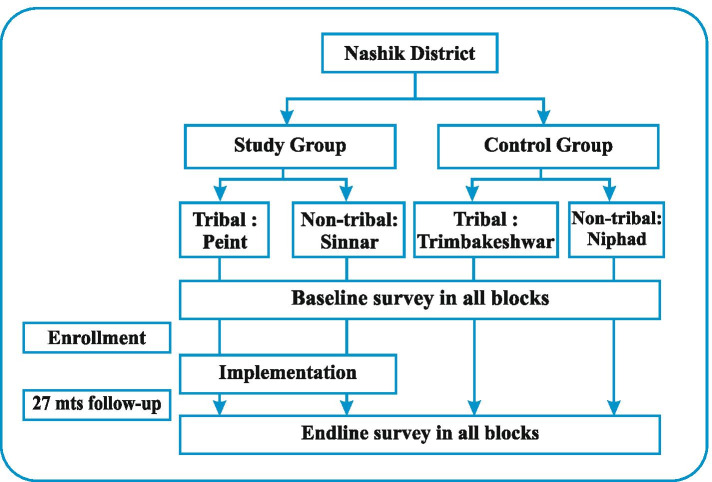
Study settings and design

**Fig. 2:**
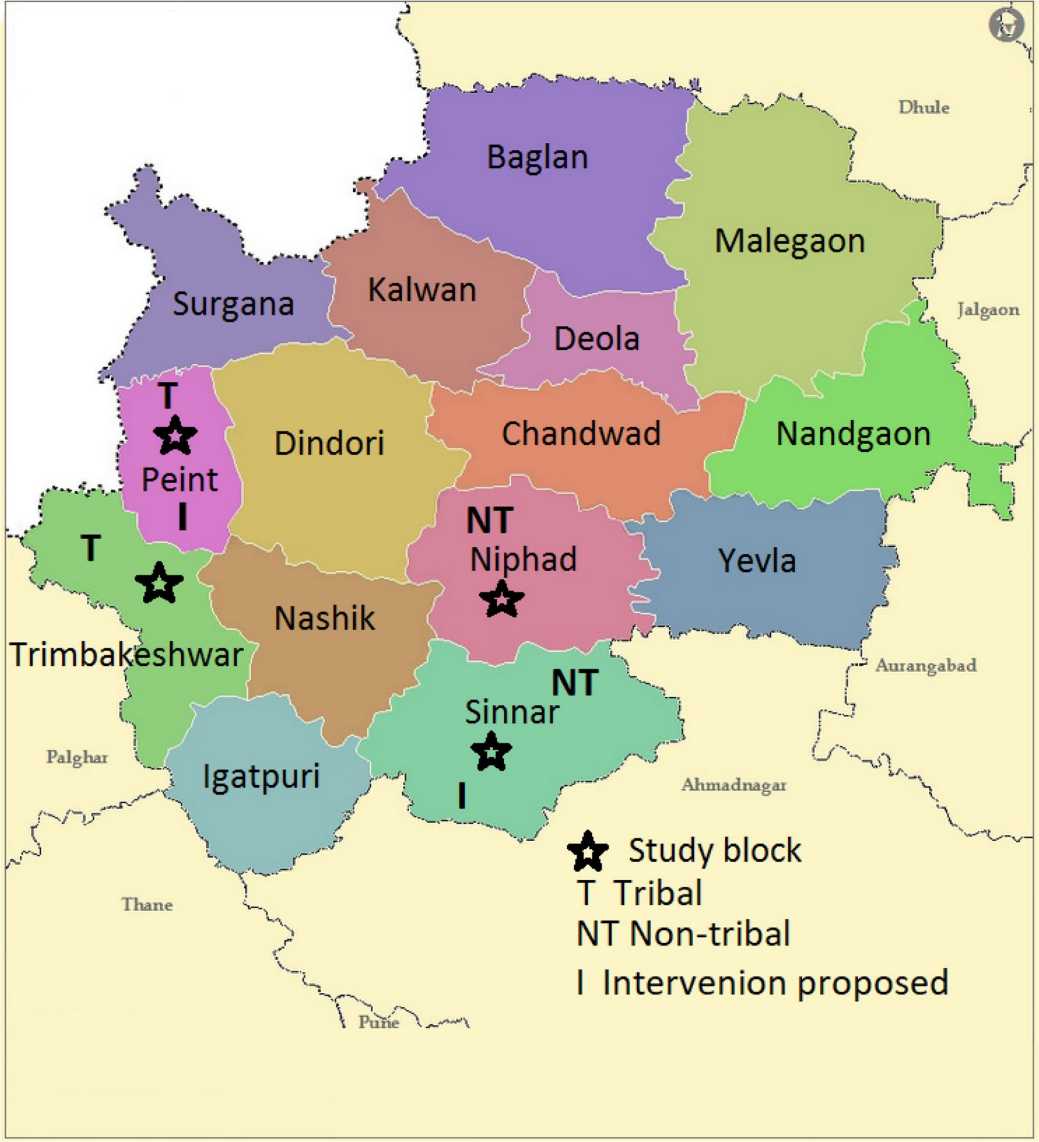
Selected blocks in Nashik district, India, 2017-18

The study group included randomly selected one tribal block having a marginalised population and one non-tribal block. The adjacent one tribal and one non-tribal block were selected as the control group. The details of the setting and design are given in Fig.1. The total population of these four blocks was 1,127,902 [3]. The block-wise map of the district is given in Fig. 2. Before the implementation of the intervention, a baseline survey was conducted in all four blocks. This article presents the results of the baseline survey.

### Study period

The study was carried out in 2018–2019, whereas baseline data was collected from May to July 2018.

### Data collection

An interview schedule was validated by experts, translated in the local language (Marathi) and pre-tested. It included demographic information, pregnancy details and risk factors associated with adverse pregnancy outcomes. Authors trained the Accredited Social Health Activists (ASHA) who collected the data through house-to-house visits and Auxiliary Nurse Midwife (ANM) who supervised the activity.

### Participants

All women in the reproductive age group (15 to 49 years) in these blocks were contacted. Women who had a pregnancy outcome in the preceding 12 months, i.e., 01 April 2017 to 31 March 2018, were included in the survey. Women who could not understand Marathi, Hindi or English or were unable to respond due to psychotic illness were excluded.

### Variables

The independent variables included age, family type, education, occupation, and residence. The second type of independent variables included the following potential risk factors: parental consanguinity, pre-existing maternal illness at conception, heavy work in the last six months of pregnancy, tobacco consumption, alcohol consumption, exposure to a pesticide, domestic violence during pregnancy. The outcome variables included abortion, stillbirth, preterm birth, low birth weight (LBW), and congenital physical defect. Operational definitions used in the study are given below.

Nuclear family: A couple and their dependent children.

Joint family: A couple with their married or unmarried children and grandchildren.

Other family: A family not considered a nuclear or joint family.

Abortion: The termination of pregnancy, spontaneous or induced, before 20 weeks of gestation.

Stillbirth: A loss of a baby at or after 20 weeks of pregnancy.

Preterm: A baby born alive before completion of 37 weeks of pregnancy.

Low Birth Weight: Birth weight of less than 2500 g.

Congenital Physical Defects: Physical defects present in the child since birth or may have become apparent during early infancy.

Pre-existing maternal illness: Illnesses such as heart disease, liver disease, renal disease, hypertension, diabetes mellitus or any other chronic disorder in the woman at the time of conception and reported by the woman.

Heavy work: Performing heavy manual work, including working for the construction of roads/buildings/dams where one needs to lift ≥10 kg load from the ground repetitively or work on the farm for ≥8 h.

### Sample size estimation

The minimum sample size assuming a stillbirth rate of 1.3% with a 95% confidence interval and an accepted difference of 0.26 was 7600 pregnant women [10]. Annual pregnancy registration in the previous two years for these blocks was over 22,000, leading to the conduction of a survey covering entire rural areas of these four blocks [11].

### Data analysis

The authors compared study and control blocks as well as tribal and non-tribal blocks. The data were analysed using the Statistical Package for Social Sciences version 25.0 and STATA software version 15.1. The age data were analysed using an independent ‘t’ test after confirming normal distribution. The Chi-square test was applied wherever applicable. We used a generalized linear regression model with a log link and binomial distribution for calculating the adjusted prevalence ratio, and *p* < 0.05 was considered significant.

## Results

In the study area, 9307 (study = 4766, control = 4541; tribal = 3298, non-tribal 6009) women were participating. The non-response rate was less than 10% except for birth weight and gestational age (about 15.1 and 37.3%, respectively).

Their socio-demographic details are shown in Table 1. The mean age of women from the tribal block was 23.7 ± 3.2 years, and for the non-tribal area, it was 24.0 ± 3.2 years; the difference was statistically significant (t = 4.2; *p* < 0.001). Teenage pregnancy was reported by 6.3% of tribal women compared to 4.3% of non-tribal women. The prevalence of adverse pregnancy outcomes was: abortion in 4.1%, stillbirth in 1.7%, preterm birth in 4.1%, LBW in 13.2%, and congenital physical defect in 2.8% of the cases. Details of adverse pregnancy outcomes in various groups are given in Table 2. The exact gestational age was unavailable for 37.3% of the women.

**Table 1:** Socio-demographic profile of women in Nasik district, India, 2017-18

	Study Area		χ2, (p)	Place of Residence		χ2, (p)	Total^a^
	Study Group	Control Group		Tribal	Non-tribal		
	n= 4766 (%)	n= 4541 (%)		n= 3298 (%)	n= 6009 (%)		N=9307 (%)
**Age (n=8790)**							
15-19	232 (5.1)	207 (4.9)	9.84 (0.08)	192 (6.3)	247 (4.3)	31.35 **(<0.001)**	439 (5.0)
20-24	2674 (58.6)	2370 (56.1)		1793 (58.4)	3251 (56.8)		5044 (57.4)
25-29	1408 (30.8)	1402 (33.2)		928 (30.2)	1882 (32.9)		2810 (32.0)
30-34	206 (4.5)	209 (4.9)		119 (3.9)	296 (5.2)		415 (4.7)
35-39	38 (0.8)	32 (0.8)		31 (1.0)	39 (0.7)		70 (0.8)
40-44	9 (0.2)	3 (0.1)		6 (0.2)	6 (0.1)		12 (0.1)
**Family Type (n=8690)**							
Nuclear	840 (18.9)	883 (20.8)	5.94 (0.051)	597 (20.1)	1126 (19.7)	3.68 (0.158)	1723 (19.8)
Joint	3563 (80.1)	3305 (77.9)		2355(79.1)	4513 (79.0)		6868 (79.0)
Other	47 (1.1)	52 (1.2)		25 (0.8)	74 (1.3)		99 (1.1)
**Education (n=8719)**							
PG/ Professional	98 (2.2)	98 (2.3)	20.76 **(0.002)**	25 (0.8)	171 (3.0)	1067.8 **(<0.001)**	196 (2.2)
Graduation	176 (3.9)	237 (5.6)		43 (1.4)	370 (6.5)		413 (4.7)
HSC/ ITI	1009 (22.4)	912 (21.7)		386 (12.9)	1535 (26.8)		1921 (22.0)
SSC	1170 (25.9)	1099 (26.1)		531 (17.7)	1738 (30.4)		2269 (26.0)
7^th^ pass	860 (19.1)	738 (17.6)		671 (22.4)	927 (16.2)		1598 (18.3)
<7^th^ pass	623 (13.8)	538 (12.8)		630 (21.0)	531 (9.3)		1161 (13.3)
Illiterate	578 (12.8)	583 (13.9)		716 (23.9)	445 (7.8)		1161 (13.3)
**Occupation (n=8994)**							
Working	3195 (68.9)	2333 (53.5)	224.43 **(<0.001)**	2624 (83.4)	2904 (49.6)	985.58 **(<0.001)**	5528 (61.5)
House wife	1441 (31.1)	2025 (46.5)		521(16.6)	2945 (50.4)		3466 (38.5)

^a^Total does not match because of non-response by the participants

*PG* Post Graduate; *HSC* Higher Secondary-school Certificate; *SSC* Secondary School Certificate; *ITI* Industrial Training Institute

**Table 2:** Prevalence of adverse outcomes in Nasik district, India, 2017-18

	Study Area				Place of Residence			
		Study Group	Control Group	χ2, (p)	Tribal	Non-tribal	χ2, (p)	Total^a^
		n (%)	n (%)		n (%)	n (%)		n (%)
**Abortion**	Y	140 (2.9)	242 (5.3)	33.79 **(<0.001)**	77 (2.3)	305 (5.1)	40.64 **(<0.001)**	382 (4.1)
	N	4626 (97.1)	4299 (94.7)		3221 (97.7)	5704 (94.9)		8925 (95.9)
	T	4766 (100.0)	4541 (100.0)		3298 (100.0)	6009 (100.0)		9307 (100.0)
**Still birth**	Y	62 (1.3)	87 (2.0)	6.34 **(0.012)**	61 (1.9)	88 (1.5)	1.545 (0.214)	149 (1.7)
	N	4564 (98.7)	4212 (98.0)		3160 (98.1)	5616 (98.5)		8776 (98.3)
	T	4626 (100.0)	4299 (100.0)		3221 (100.0)	5704 (100.0)		8925 (100.0)
**Preterm birth**	Y	141 (4.5)	101 (3.7)	2.67 (0.102)	48 (2.7)	194 (4.8)	12.73 **(<0.001)**	242 (4.1)
	N	2961 (95.5)	2636 (96.3)		1712 (97.3)	3885 (95.2)		5597 (95.9)
	T	3102 (100.0)	2737 (100.0)		1760 (100.0)	4079 (100.0)		5839 (100.0)
**Low birth weight**	Y	538 (12.9)	502 (13.4)	0.47 (0.492)	458 (17.6)	582 (11.0)	66.06 **(<0.001)**	1040 (13.2)
	N	3626 (87.1)	3232 (86.6)		2148 (82.4)	4710 (89.0)		6858 (86.8)
	T	4164 (100.0)	3734 (100.0)		2606 (100.0)	5292 (100.0)		7898 (100.0)
**Congenital physical defect**	Y	116 (2.5)	129 (3.0)	2.07 (0.15)	94 (2.9)	151 (2.7)	0.567 (0.451)	245 (2.8)
	N	4484 (97.5)	4138 (97.0)		3106 (97.1)	5516 (97.3)		8622 (97.2)
	T	4600 (100.0)	4267 (100.0)		3200 (100.0)	5667 (100.0)		8867 (100.0)

^a^Total does not match because of non-response by the participants; Y-yes, N-no, T-total

Prevalence of consanguinity, heavy work during the last six months of pregnancy, tobacco consumption, alcohol consumption, direct exposure to pesticides, domestic violence during pregnancy and pre-existing illness were 18.5, 18.7, 5.6, 0.5, 2.3, 0.8, and 2.2% respectively. The distribution of these risk factors in various groups is given in Table 3.

**Table 3:** Potential risk factors during pregnancy and residence in Nasik district, India, 2017-18

		Study Area			Place of Residence			
		Study Group n (%)	Control Group n (%)	χ2, (p)	Tribal n (%)	Non-tribal n (%)	χ2, (p)	Total^a^
**Consanguinity**	Y	794 (17.4)	848 (17.4)	8.12 **(0.004)**	651 (21.2)	991 (17.1)	22.3 **(<0.001)**	1642 (18.5)
	N	3771 (82.6)	3446 (82.6)		2418 (78.8)	4799 (82.9)		7217 (81.5)
**Heavy work in last 6 months**	Y	708 (16.2)	869 (21.3)	36.57 **(<0.001)**	882 (31.5)	695 (12.3)	453.6 **(<0.001)**	1577 (18.7)
	N	3664 (83.8)	3206 (78.7)		1919 (68.5)	4951 (87.7)		6870 (81.3)
**Tobacco**	Y	234 (4.9)	283 6.2)	7.64 **(0.006)**	406 (12.3)	111 (1.9)	443.4 **(<0.001)**	517 (5.6)
	N	4523 (95.1)	4257 (93.8)		2892 (87.7)	5888 (98.1)		8780 (94.4)
**Alcohol**	Y	26 (0.6)	18 (0.4)	1.15 (0.283)	27 (0.8)	17 (0.3)	12.5 **(<0.001)**	44 (0.5)
	N	4668 (99.4)	4488 (99.6)		3271 (99.2)	5885 (99.7)		9156 (99.5)
**Exposure to pesticides**	Y	78 (1.6)	131 (2.9)	16.57 **(<0.001)**	93 (2.8)	116 (1.9)	7.8 **(<0.001)**	209 (2.3)
	N	4671 (98.4)	4389 (97.1)		3184 (97.2)	5876 (98.1)		9060 (97.7)
**Domestic violence**	Y	32 (0.7)	42 (0.9)	1.90 (0.167)	48 (1.5)	26 (0.4)	25.6 **(<0.001)**	74 (0.8)
	N	4683 (99.3)	4445 (99.1)		3239 (98.5)	5889 (99.6)		9128 (99.2)
**Existing illness**	Y	95 (2.0)	109 (2.4)	1.57 (0.209)	40 (1.2)	164 (2.8)	23.9 (**<0.001)**	204 (2.2)
	N	4591 (98.0)	4409 (97.6)		3258 (98.8)	5742 (97.2)		9000 (97.8)

^a^Total does not  match because of non-response by the participant; Y-yes, N-no

Significant differences were present between the study and control group for some of the risk factors, but the distributions of all risk factors between tribal and non-tribal areas differed profoundly. Except for pre-existing illness, all the other risk factors occurred more frequently among women from the tribal areas.

Table 4 shows the adjusted prevalence ratio of the adverse pregnancy outcomes and the risk factors after multivariate analysis. All the eight potential risk factors were adjusted in the multivariate analysis to evaluate their effect on each of the adverse pregnancy outcomes. Performing heavy work in the last 6 months of pregnancy was a risk factor for all the adverse outcomes except stillbirth. Pre-existing maternal illness was the second common risk factor that was associated with abortion, stillbirth and LBW. Parental consanguinity and tobacco consumption were associated with stillbirth and congenital physical defects. Alcohol consumption, exposure to pesticides, and domestic violence didn’t influence any outcome.

**Table 4:** Adverse pregnancy outcomes and potential risk factors in Nasik district, India, 2017-18

		Abortion^a^		APR (95% CI) ***p***-value	Still birth^a^		APR (95% CI) ***p***-value	Preterm birth^a^		APR (95% CI) ***p***-value	Low birth weight^a^		APR (95% CI) ***p***-value	Congenital physical defect^a^		APR (95% CI) ***p***-value
		Y	N		Y	N		Y	N		Y	N		Y	N	
**Consanguinity**	**Y**	65	1577	1(0.8-1.4) 0.76	39	1538	1.5(1.0-2.3) **0.03**	36	950	1.0(0.7-1.4) 0.81	180	1215	0.9(0.8-1.1) 0.41	76	1488	1.9(1.4-2.6) **<0.001**
	**N**	302	6915		102	6813		200	4483		819	5341		148	6726	
**Heavy work in last 6 mths.**	**Y**	92	1485	1.9(1.5-2.5) **<0.001**	37	1438	1.4(0.9-2.1) 0.1	48	924	1.4(1.1-2.0) **0.04**	211	1028	1.3(1.1-1.5) **0.004**	65	1403	1.8(1.3-2.5) **0.001**
	**N**	257	6613		102	6511		182	4336		726	5279		150	6423	
**Tobacco**	**Y**	10	507	0.5(0.3-1.0) 0.06	18	489	2.1(1.2-3.6) **0.01**	16	311	1.5(0.9-2.6) 0.13	73	345	1.2(0.9-1.5) 0.23	26	473	2.2(1.4-3.4) **0.001**
	**N**	371	8409		131	8278		226	5279		964	6509		219	8140	
**Alcohol**	**Y**	1	43	0.5(0.1-3.7) 0.53	2	41	1.5(0.4-5.9) 0.59	2	22	1.6(0.4-6.4) 0.5	7	26	1.2(0.6-2.4) 0.56	1	41	0.5(0.1-3.8) 0.53
	**N**	370	8786		144	8642		239	5507		1022	6765		243	8488	
**Exposure to pesticides**	**Y**	10	199	1(0.5-2.0) 0.95	4	195	0.8(0.3-2.6) 0.76	4	98	0.9(0.4-2.4) 0.85	28	149	1.0(0.7-1.5) 0.9	17	181	1.4(0.7-2.9) 0.32
	**N**	371	8689		144	8545		238	5473		1005	6688		226	8406	
**Domestic violence**	**Y**	2	72	0.6(0.2-2.5) 0.52	1	71	0.5(0.8-3.9) 0.54	2	43	1.0(0.2-3.9) 0.98	11	41	0.9(0.5-1.8) 0.84	6	66	2.0(0.9-4.9) 0.11
	**N**	362	8766		144	8622		238	5493		1019	6754		237	8472	
**Existing illness**	**Y**	31	173	3.1(2.0-4.9) **<0.001**	11	162	3.0(1.4-6.4) **0.004**	7	104	1.0(0.4-2.7) 0.97	35	115	1.8(1.3-2.5) **0.001**	5	162	1.4(0.6-3.3) 0.46
	**N**	344	8656		138	8518		230	5415		998	6661		239	8365	
**Residence in tribal area**	**Y**	77	3221	0.5(0.4-0.7) **<0.001**	61	3160	1.1(0.7-1.6) 0.71	48	1712	0.6(0.4-0.8) **0.001**	458	2148	1.5(1.3-1.7) **<0.001**	94	3106	0.7(0.5-0.9) **0.02**
	**N**	305	5704		88	5616		194	3885		582	4710		151	5516	

^a^Total does not match because of non-response by the participants; APR- Adjusted Prevalence Ratio, Y-yes, N-no

## Discussion

This study was designed to compare the distribution of potential risk factors in consideration of adverse pregnancy outcomes among women from tribal- and non-tribal areas in India. We found a difference in the distribution of risk factors like parental consanguinity, heavy maternal work, tobacco consumption and exposure to pesticides during pregnancy in both groups; however, all risk factors except pre-existing maternal illness were more common in the tribal area. Similarly, differences existed for ceratin pregnancy outcomes in the study and control group, as well as in the tribal and non-tribal groups. Risk factors like parental consanguinity, pre-existing illness, heavy work in the last six months and alcohol consumption were identified as risk factors for adverse pregnancy outcomes.

The significant differences in the distribution of some variables in the tribal and non-tribal areas may be due to the sheer large number of participants. In India, a National Survey showed a high prevalence of consanguineous marriages in Maharashtra and Southern States, ranging from 28 to 38% [12]. However, the prevalence observed in the present study is lower than the reported range of 20.3 to 36% from other studies [13–15]. The lower prevalence in the present study may be due to the overall improvement in education in recent years.

Farming is a major occupation in rural and tribal parts of India, where even pregnant women are indulged in performing heavy manual work. Many such women also work at construction sites on daily wages, where they repeatedly lift heavy loads. Lifting such heavy loads can increase the risk of abortion, especially after the first trimester [16]. Our study analyzed heavy work as a risk factor to estimate the effect on both abortion and stillbirth. Overall, 18.7% of women performed heavy work during the last six months of pregnancy; however, reported more frequently in tribal women.

The study observed less frequent tobacco consumption during pregnancy than other studies from India [17, 18]. The most common form of tobacco consumption was found for Mishri (roasted tobacco) on gums and teeth. Alcohol consumption in Indian women is less frequent than in the western world and generally rare during pregnancy [19]. In the present study, overall alcohol consumption during pregnancy was lower than reported in a study from Karnataka, India [13]. However, as previously observed, it was higher in tribal women [20].

Pesticide spraying on grapes is a highly prevalent seasonal work in the Nashik district, but women are occasionally engaged. The association of pesticides with adverse pregnancy outcomes is inconsistent and depends on the type of pesticide, duration/amount of exposure and gestational age at that time [21–23]. We found no association with adverse pregnancy outcomes in the present study.

Various studies have reported the prevalence of domestic violence during pregnancy to range from 7.1–18%, far higher than we report herein in our study [24, 25]. However, Indian women are reluctant to divulge such information, particularly when the interviewer is known to the husband. So these figures may be just “the tip of the iceberg”. The pre-existing maternal illness at conception, like endocrine abnormalities, heart disease, etc., affects maternal health and pregnancy outcomes [26–28]. In the present study, 2.2% of the women had reported pre-existing illnesses; however, there is a dearth of information about the prevalence of pre-existing illness in pregnant women in the community. Pre-existing maternal illness was more in non-tribal areas, probably due to better availability and accessibility of health care services in the non-tribal area leading to more detection.

### Abortion

In the present study, 4.1% of women reported abortion, consistent with the Indian estimate of 4.7% [29]. Performing hard manual work, including lifting heavy weights during early pregnancy, a known risk factor for abortion [16], is once again confirmed in this study. Analogous with other studies, pre-existing maternal illness had three times increased risk of abortion [27, 30]. The lower abortion rate in the tribal area may be due to lack of access to the pregnancy termination facilities and affordability; however, we did not differentiate between spontaneous and induced abortion. It was not consistent with the study in the rural and tribal communities from Maharashtra, India [31]. Some studies observed an association between consanguineous marriage and abortion which was not found in our study [13, 14]. Studies have shown an association of alcohol consumption during pregnancy, especially the first trimester, with abortions [32, 33] but similar to the systematic review [34], which we cannot confirm herein.

### Stillbirth

The stillbirth rate that we found was 1.7%, which is similar to a study across Maharashtra and other states of India [35, 36]. Consanguineous marriage and tobacco consumption were identified as significant risk factors for stillbirth, which is consistent with other studies [13, 37–39]. A meta-analysis reported a dose-response effect of maternal smoking during pregnancy on stillbirth [40]. Studies have identified alcohol consumption during pregnancy as a risk factor for stillbirth [41, 42]. We are not able to confirm these findings, probably because of the small number of women consuming alcohol in our study. Similar to the present study, pre-existing maternal illnesses like diabetes mellitus or thyroid dysfunction have been associated with stillbirth [26, 27]. Some studies observed an association between domestic violence and stillbirth, which was not seen in this study [24, 43].

### Preterm birth

The present study reported a preterm birth rate of 4.1%, which is lower than it has previously been reported (9–18%) in Indian studies [44, 45], as well as the global estimate of 10.6% [46]. However, these Indian studies are either past studies or not from progressive states. The contributing reasons may be the recall bias and inability of many mothers (overall 37.26 and 46.63% from tribal area) to report the exact gestation.

Parental consanguinity has been associated with preterm birth in previous studies; however, it was not observed in the present study [47, 48]. A study from Denmark reported a notably higher risk for preterm births with lifting heavy loads [49]. A study from the Netherlands did not find consistent significant associations between physically demanding work and preterm delivery or LBW [50]. In the present study, heavy work during the last six months of pregnancy was identified as a risk factor for preterm birth.

The association of tobacco consumption or smoking during pregnancy with preterm birth is due to various obstetric factors, and the risk is shown to increase with the amount of smoke [51, 52]. The role of alcohol consumption in preterm birth is controversial [34, 53]. Few studies have identified exposure to pesticides as a risk factor for preterm birth; however, findings are not consistent [21, 54, 55]. A meta-analysis reported a 46% risk of preterm birth in women exposed to domestic violence during pregnancy [56]. Pre-existing maternal illnesses like diabetes mellitus or liver disorder are associated with preterm birth [26, 30, 57]. In the present study, these risk factors were not associated with preterm birth.

### Low birth weight

The 13.2% LBW rate that we found is almost similar to the range of 11.6 to 16.4% that has been reported in other Indian studies [58, 59]. Performing heavy work during the last six months of pregnancy was associated with LBW, similar to another study [60]. Tobacco, in any form, has been associated with LBW [17, 51]. During pregnancy, exposure to various pesticides has been identified as a risk factor for LBW; however, these results seem to be inconsistent [21, 22]. The association depends on various factors like the type of pesticide, gestational period, duration and amount of exposure.

In a previous study, the risk of LBW in women with a chronic illness during pregnancy was five times higher than in healthy subjects, far higher than we found in our study [61]. A systematic review reported domestic violence as a risk factor for LBW; however, it is not observed in this study [56]. LBW was significantly higher in the tribal area, which stands in accordance with a study from another district of India [31]. It reflects the effects of factors like maternal age, education, nutrition, Antenatal care (ANC) visits, availability and accessibility of healthcare facilities etc.

### Congenital physical defect

We observed a slightly higher rate of congenital physical defects than the national estimate [62]. Similar to the previously published studies, we found consanguinity as a risk factor for a fetal congenital physical defect [37, 63]. The proportion of congenital physical birth defects may be reduced by creating awareness about the effects of consanguineous marriage. This intervention requires minimal resources and could have a significant benefit for the outcome of the pregnancy. We also found that heavy work during the last six months of pregnancy and tobacco consumption were both associated with a congenital physical defect. A meta-analysis reported an association of maternal smoking with congenital oro-facial clefts [64], whereas another systematic review reported many other birth defects in the infants [65]. Alcohol consumption during pregnancy [66, 67], and pre-existing illness in pregnant women have been associated with congenital physical defects [63, 68] but were not found in our dataset.

### Strengths and limitations

Our study involved more than nine thousand women from four different blocks in the area; it included various adverse pregnancy outcomes, as well as various risk factors. The study was conducted with the help of existing health care workers. Nevertheless, the overall findings may not apply to the general community because 35% of the women in the study were from tribal areas. In this survey-oriented research, over 37% of women did not remember the gestational age because all were from rural areas, including many from tribal areas, which led to a recall bias, being a major limitation of this study. Details regarding pre-existing illnesses were not adequately studied. Moreover, we were unable to study further details in the case of abortion.

## Conclusion

This large cross-sectional study from India identified risk factors, such as parental consanguinity, pre-existing maternal illness at conception, tobacco consumption and heavy work in the last six months of pregnancy, which were associated with one or more adverse outcomes during pregnancy. As most of these risk factors were behaviourally related, there is a need to emphasize on maternal behaviour during the preconception phase and antenatal care.

## Data Availability

Data used in the analysis are available from the corresponding author on reasonable request.

## Abbreviations

PCC: Preconception care
INAP: India Newborn Action Plan
ASHA: Accredited Social Health Activists
ANM: Auxiliary Nurse Midwife
ARR: Adjusted risk ratio
LBW: Low birth weight
SSC: Secondary School Certificate
IHDS: India Human Development Survey
ANC: Antenatal care
PG: Post Graduate
HSC: Higher Secondary-school Certificate
ITI: Industrial Training Institute
